# The role of neurotransmitter systems in mediating deep brain stimulation effects in Parkinson’s disease

**DOI:** 10.3389/fnins.2022.998932

**Published:** 2022-10-05

**Authors:** Faisal Alosaimi, Jackson Tyler Boonstra, Sonny Tan, Yasin Temel, Ali Jahanshahi

**Affiliations:** ^1^Department of Neurosurgery, Maastricht University Medical Centre, Maastricht, Netherlands; ^2^Department of Physiology, Faculty of Medicine, King Abdulaziz University, Rabigh, Saudi Arabia; ^3^Netherlands Institute for Neuroscience, Royal Netherlands Academy of Arts and Sciences, Amsterdam, Netherlands

**Keywords:** deep brain stimulation, DBS, neurotransmitters, Parkinson’s disease, PD

## Abstract

Deep brain stimulation (DBS) is among the most successful paradigms in both translational and reverse translational neuroscience. DBS has developed into a standard treatment for movement disorders such as Parkinson’s disease (PD) in recent decades, however, specific mechanisms behind DBS’s efficacy and side effects remain unrevealed. Several hypotheses have been proposed, including neuronal firing rate and pattern theories that emphasize the impact of DBS on local circuitry but detail distant electrophysiological readouts to a lesser extent. Furthermore, ample preclinical and clinical evidence indicates that DBS influences neurotransmitter dynamics in PD, particularly the effects of subthalamic nucleus (STN) DBS on striatal dopaminergic and glutamatergic systems; pallidum DBS on striatal dopaminergic and GABAergic systems; pedunculopontine nucleus DBS on cholinergic systems; and STN-DBS on locus coeruleus (LC) noradrenergic system. DBS has additionally been associated with mood-related side effects within brainstem serotoninergic systems in response to STN-DBS. Still, addressing the mechanisms of DBS on neurotransmitters’ dynamics is commonly overlooked due to its practical difficulties in monitoring real-time changes in remote areas. Given that electrical stimulation alters neurotransmitter release in local and remote regions, it eventually exhibits changes in specific neuronal functions. Consequently, such changes lead to further modulation, synthesis, and release of neurotransmitters. This narrative review discusses the main neurotransmitter dynamics in PD and their role in mediating DBS effects from preclinical and clinical data.

## Introduction

Deep brain stimulation (DBS) involves a stereotaxic electrode implantation into a specific brain region (Benabid et al., 1993) and has been successful in managing symptoms in several movement disorders including Parkinson’s disease (PD) (Wichmann and DeLong, 2006). However, the exact mechanisms behind DBS effects and side effects in PD are not fully understood. Several theories work to explain underlying mechanisms of DBS, the most common being the rate and pattern theories. These theories propose DBS modulates an overactive basal ganglia target in PD patients by normalizing the firing rate at the electrophysiological single-cell level (Agnesi et al., 2013; Ashkan et al., 2017), interferes with pathological patterns such as subcortical beta oscillations (13–30 Hz), promotes cortical gamma activity (40–200 Hz), and modulates local field potential readouts (Eusebio and Brown, 2009; Cheney et al., 2013; Muthuraman et al., 2021). However, those theories only involve effects of DBS on local, and to a lesser extent, remote readouts. For instance, electrophysiological recordings of neuronal activity show that ventroanterior (VA) and ventrolateral (VL) thalamus-DBS (10 Hz) suppress the beta oscillation and increase the gamma power in the motor cortex in both *in vitro* and *in*vivo animal models and improves motor function (Tucker et al., 2019). In addition, subthalamic nucleus (STN) DBS leads to a short-latency excitatory effect that tonically increases the firing rate in the globus pallidus internal/external (GPi/GPe) followed by post-stimulation suppression of the firing rate (Hashimoto et al., 2003). Another study showed STN-DBS only suppressed the neuronal activity in the GP and substantia nigra pars reticulata (SNr) in anesthetized rats (Benazzouz et al., 1995; Beurrier et al., 2001). Despite this discrepancy, another explanation could be from variations between DBS local effects vs. orthodromic activations from the STN to the GPe and GPi as well as the motor cortex. Furthermore, preclinical studies show that low-frequency stimulation (LFS) of the pedunculopontine nucleus (PPN) suppresses the firing rate in the STN and SNr in PD rat models (Alam et al., 2012; Park et al., 2014).

Neurotransmitters are largely neglected when discussing mechanisms behind DBS possibly due to technical challenges of recording real-time changes in remote areas distant from the stimulation zone. Both clinical and preclinical PD data show DBS modulates several neurotransmitter networks such as dopaminergic, glutamatergic, GABAergic, serotonergic, and cholinergic systems (Ogura et al., 2004; Boulet et al., 2006; Temel et al., 2007; He et al., 2014; Wen et al., 2015). For example, the effect of STN-DBS on motor function could be associated with various effects on striatal dopaminergic and glutamatergic neurotransmission (Boulet et al., 2006; He et al., 2014), while the effect of GPi-DBS could be associated with changes in the striatal dopaminergic and pallidum GABAergic systems (Ogura et al., 2004; Xiao et al., 2019). Likewise, the PPN may be investigated as a new candidate DBS target to assess its effects on the cholinergic system to reduce gait disturbances in PD (Wen et al., 2015). Additionally, STN-DBS has been associated with mood-related side effects possibly linked to brainstem serotoninergic systems (Temel et al., 2007). STN-DBS therapeutic effects showed to be diminished by severe degeneration of the locus coeruleus (LC) noradrenergic system (Guimarães et al., 2013; Faggiani et al., 2015). Furthermore, DBS has shown to modulate glia cells to induce glutamate release and modulate neurotransmission in nearby neurons in PD (McGeer and McGeer, 2004, 2008; Vedam-Mai et al., 2012).

Deep brain stimulation has also shown to exhibit long-term effects possibly due to neuroplasticity involving transmitter levels and neuronal communication (Ashkan et al., 2017). Understanding patterned changes at the transmitter level will contribute to a better understanding of the underlying mechanisms of DBS and lead to optimized targeting, improved symptom management, lesser side effects, and an improved quality of treatment. In this article, we discuss neurotransmitter dynamics in PD and briefly revise current theories on the mechanisms behind DBS within the context of neurotransmitter dynamics. We then address the extent of alterations in neurotransmitter systems before and after DBS in PD, the circuits involved, and the impacts such changes can have on PD symptoms.

## Neurotransmitter systems’ dynamics in Parkinson’s diseases

Parkinson’s disease (PD) has been pathologically categorized as a disorder of the basal ganglia, a network consisting of three main regions, (1) the striatum (caudate and lentiform nuclei); the lentiform nucleus made up of putamen and dorsal pallidum; dorsal pallidum consists of GPi and GPe, (2) the nigra complex (SNc and SNr), and (3) the STN (Jahanshahi et al., 2020). The neocortex, thalamus and brainstem regions such as PPN are also heavily involved in PD as they are connected to the basal ganglia at multiple levels (Jahanshahi et al., 2020). The Albin et al. (1989)–DeLong (1990) model describes how the basal ganglia regulates movement execution or inhibition by two main pathways (Albin et al., 1989; DeLong, 1990); (1) The direct pathway where the striatum projects GABAergic inhibitory activity directly to the GPi/SNr that blocks inhibitory output to the thalamus; the thalamus projects excitatory glutamatergic activity to the cortex allowing for movement execution, and (2) the indirect pathway where the striatum projects GABAergic inhibitory activity to the GPe and STN activating the GABAergic inhibition of the GPi/SNr to the thalamus; thalamic glutamatergic activity is inhibited and ultimately suppresses cortical activity. Moreover, the striatum also receives dopaminergic projections as an output signal from the SNc that regulates both direct and indirect routes (Albin-Delong classic model). In addition, Nambu et al. (2002) described the “hyper-direct” pathway, a fast pathway forming a loop from the cortex that has a glutamatergic excitatory connection to the GPi via the STN and stimulates the GPi which then inhibits the thalamus and neocortex (Nambu et al., 2002).

The main pathological hallmark of PD is the neurodegeneration of SNc dopaminergic neurons (Yelnik, 2002). Dopaminergic neurons from the SNc that innervate the striatum are from the nigrostriatal pathway that regulates both the direct and indirect pathways. The nigrostriatal pathway connects to the thalamus and cortex via striatal interneurons and spinal projection neurons that have dopamine D1 or D2 receptors. Dopamine medications for PD reduce symptoms via their action on D1 receptors but have inhibitory effects on D2 receptors that could partially underlie side effects. This is one reason why medication such as Levodopa combined with D2 receptor antagonist increases the efficacy of the medication (Sarkar et al., 2016).

Degeneration of dopaminergic SNc neurons causes dysfunction of the direct and indirect pathways in the basal ganglia leading to less inhibition of the GPe and STN, increased activity of GPi, and decreased overall excitation input from the thalamus to the cortex. Furthermore, the STN also receives afferent glutamatergic innervations from the cortex and the parafascicular nucleus of the thalamus (Kitai and Deniau, 1981; Mouroux et al., 1995). Direct glutamatergic neuronal connections from the motor cortex (MC) to the STN investigated in 6-hyrdoxydopamine (6-OHDA) hemi-lesioned rats using anterograde tracing (Wang et al., 2018) showed MC-STN connectivity was impaired and glutamatergic terminals were reduced (Wang et al., 2018). In addition, the STN receives GABAergic input from the GPe, dopaminergic input from the SNc, and cholinergic/glutamatergic input from the PPN (Nauta and Cuenod, 1982; Campbell et al., 1985; Lavoie and Parent, 1994). As mentioned above, this dysfunctional connection of the MC-STN could lead to overactive STN glutamatergic neurons that may produce excitotoxins projecting to the SNc and GPi (Rodriguez et al., 1998; Przedborski, 2005; Dexter and Jenner, 2013). Glutamatergic excitotoxicity was found to be related in several validated animal models of PD, such as 6-OHDA and 1-methyl-4-phenyl-1,2,3,6-tetrahydropyridine (MPTP) (Dexter and Jenner, 2013). Furthermore, this excitotoxicity can be inhibited by a glutamate antagonist MK801 but not with the GABA antagonist bicuculline (Wu et al., 2012).

## Role of neurotransmitters in mediating deep brain stimulation effects

Historically, surgical treatment using vascular or chemical lesions of the GP or thalamus were used to reduce PD symptoms before the discovery of Levodopa in 1967 (Quinn, 1999; Olanow, 2002). However, these treatments failed to suppress disease progression and lead to several adverse effects such as dyskinesia, motor fluctuations, and drug-resistance (Marsden and Parkes, 1977). The development of stereotactic surgery, neuroimaging, and intraoperative electrophysiology enabled more accurate brain region targeting with a higher therapeutic yield and less overall harm (Benabid et al., 1993; Benabid et al., 2001). STN-DBS was done in 1993 by Benabid then became the standard treatment especially for refractory PD patients (Benabid et al., 2001).

Despite progress in DBS as a treatment for movement disorders, its exact mechanisms of action have not been fully unraveled. Several theories have been proposed, the most common being the neuronal firing “rate-model” and the patterned “synchronized oscillations” hypotheses (Bergman et al., 1994; Levy et al., 2000; Brown et al., 2001; Breit et al., 2004; Lozano and Eltahawy, 2004; Benabid, 2007; Rosa et al., 2012; Ashkan et al., 2017). The rate model hypothesis postulates DBS blocks overactive basal ganglia neuronal firing rates in the STN and GPi caused by PD pathology. The blocking of the overactive basal ganglia was predicted by lesion effects of earlier neuromodulation techniques (Quinn, 1999). Interestingly, a post-mortem study on spinocerebellar ataxia disease found a consistent degeneration of SNc, although patients did not exhibit parkinsonian symptoms (Schöls et al., 2015). Further histological analysis of this post-mortem brain tissue showed a significant lesion in brain regions such as the thalamic ventral anterior and ventral lateral nuclei, pallidum, PPN, and STN.

Consequently, these findings suggest that lesion effects of these targets lead to a therapeutic output on PD symptoms. The pattern “synchronized oscillations” hypothesis has also been proposed as a mechanism of DBS, where pathological beta-band oscillatory activity (abnormal synchronized bursts of activity) in loops between the cortex, basal ganglia, thalamus, PPN, and cerebellum contribute to the genesis of PD motor symptoms (Bevan et al., 2006). DBS is thought to disrupt and suppress pathological beta-band oscillations and promote gamma power, thereby reducing bradykinesia and rigidity symptoms (Muthuraman et al., 2021).

In addition to local effects, DBS has shown to have downstream effects on remote areas. For instance, STN-DBS in PD has led to increased striatal dopamine release in PD rats (Meissner et al., 2001, 2002; He et al., 2014; Yamamoto et al., 2014). In addition, another study found a correlation between an increase in striatal monoamine (MA) levels and STN beta activity suppression via STN-DBS in a rat model of PD (hemi-PD 6-OHDA) (Yamamoto et al., 2014). GPi-DBS has also been shown to stimulate striatal dopamine release in rodents (Dostrovsky et al., 2000; Johnson and McIntyre, 2008). Moreover, preclinical research shows STN-DBS reduces dorsal raphe nucleus (DRN) serotonergic neuron firing rates in both PD and healthy control rat models (Temel et al., 2007) while PPN-DBS reduces acetylcholine (ACh) loss in the ventral thalamus (Wen et al., 2015).

**FIGURE 1:**
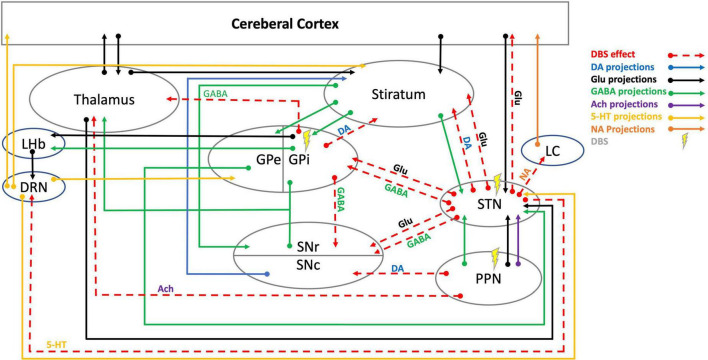
Schematic illustration of DBS neurotransmitter mediation in cortico-thalamic-basal ganglia-brainstem circuits. 5-HT, 5-hydroxytryptamine (serotonin); Ach, acetylcholine; DA, dopamine; DBS, deep brain stimulation; DRN, dorsal raphe nucleus; GABA, γ-aminobutyric acid; Glu, glutamate; GPe, globus pallidus external; GPi, globus pallidus internal; LC, locus coeruleus; LHb, lateral habenula; NA, noradrenaline; PPN, pedunculopontine nucleus; SNc, substantia nigra pars compacta; SNr, substantia nigra pars reticulata, STN, subthalamic nucleus. DBS effects data are cited in Table 1.

Research suggests DBS has multimodal effects that are not simply due to an inhibition of local axons, but from effects that may pass through fiber, efferent, and afferent axons through orthodromic and antidromic pathways (McIntyre et al., 2004; Vachez and Creed, 2020). Moreover, DBS is thought to exhibit long-term effects, including tyrosine hydroxylase (TH) cell survival in the striatum as a possible form of neurogenesis and neuroplasticity (Khaindrava et al., 2011). Additionally, alleviating pathological oscillations by continuous DBS showed short-term and long-term synaptic plasticity in the SNr (Milosevic et al., 2018, 2019). These long-term effects may be activated via neurochemical changes displayed through neurotransmitters’ levels and neuronal communications. The following section will extensively discuss dynamic changes in main neurotransmitter systems involved in PD that mediate DBS effects (Table 1, Figure 1).

**TABLE 1:** Neurotransmitters dynamics in response to DBS treatment in PD.

NT	DBS target	Study type	Main findings	References
Dopamine	STN	*In vivo* rat model	Increased striatal dopamine release and improved motor symptoms.	He et al., 2014
Dopamine	STN	*In vivo* rat model	Increased striatal dopamine release.	Yamamoto et al., 2014
Dopamine	STN	*In vivo* rat model	Increased striatal dopamine metabolites in awake, freely moving animal.	Meissner et al., 2002
Dopamine	STN	*In vivo* rat model	Increased expression of DR1 and decrease DR2 and DR3 in the striatum.	Carcenac et al., 2015; Melon et al., 2015
Dopamine	STN	*In vivo* rat model	Increased extracellular dopamine release in the striatum.	Walker et al., 2009
Dopamine	STN	*In vivo* rat model	Increased and decreased the spiking activity of SNc neurons.	Sahai et al., 2020
Dopamine	STN	*In vivo* rat model	Increased striatal dopamine release.	Du et al., 2018
Dopamine	STN	*In vivo* rat model	Long-term effects on survival of striatum dopaminergic cells and cell proliferation in HIPP and olfactory bulb.	Khaindrava et al., 2011
Dopamine	STN	*In vivo* monkey model	Induced phasic DA release in striatum according to *in vivo* fast-scan cyclic voltammetry.	Nakajima et al., 2017
Dopamine	STN	*In vivo* rhesus macaque model	Increased striatal dopamine release. The level of dopamine release was depended on precision of stimulation site.	Min et al., 2016
Dopamine	STN	*In vivo* Pig model	Increased striatal dopamine release depended on stimulation intensities and frequencies.	Shon et al., 2010
Dopamine	STN	Human clinical trail	PET imaging showed a decrease of VMAT2 transporter in the striatal reflecting a potential increase of dopamine levels.	Smith et al., 2019
Dopamine	STN	Human clinical trail	Increased CSF catecholamine levels and tittered down Levodopa with an improved motor function.	Yamamoto et al., 2015
Dopamine	STN	Human clinical trail	No changes in DAT availability and an increase in DR2 binding were detected in the striatal.	Hesse et al., 2008
Dopamine	STN	Human clinical trail	RacloBP that reflects DR2/DR3 density and/or synaptic dopamine levels in the striatal were reduce after STN-DBS.	Thobois et al., 2010
Dopamine	STN	Human clinical trail	Induced the stabilization of synaptic dopamine concentrations in the striatal.	Nimura et al., 2005
Dopamine	STN	Human clinical trail	PET imaging showed no evidence of increased striatal dopamine concentration under effective STN-DBS in humans.	Hilker et al., 2003
Dopamine	STN	Human clinical trail	PET imaging showed no evidence of increased striatal dopamine concentration.	Strafella et al., 2003
Dopamine	GPi	*In vivo* rat model	No change was found in the striatal dopaminergic metabolism in naïve and 6-OHDA lesioned rats using microdialysis.	Meissner et al., 2001
Dopamine	GPi	*In vivo* rat model	The striatal dopamine concertation increases and neural firing decrease after GPi-DBS using microdialysis.	Xiao et al., 2019
Dopamine	GPi	Human clinical trail	Increase pallidal dopamine but no correlation with improvement in rigidity.	Martinez et al., 2013
Glutamate	STN	*In vivo* rat model	STN glutamate level rapidly elevated during stimulation, sustained during stimulation, and descended slowly toward the baseline after stimulation.	Lee et al., 2007
Glutamate	STN	*In vivo* rat model	Glutamate levels increased in SNr with both intact and hemiparkinsonian rats.	Boulet et al., 2006
Glutamate	STN	*In vivo* rat model	Chronic (5 week) STN-DBS of 6-OHDA freely moving rats restored normal levels of glutamate metabolite in the striatum using *in vivo* 11.7 tesla MRS.	Chassain et al., 2016
Glutamate	STN	*In vivo* rat model	Increased extracellular glutamate levels in SNr and GPi in both PD and control.	Windels et al., 2000
Glutamate	STN	*In vivo* rat model	Downregulated of CaMKIIa and Homer1 genes that are involved in glutamate neurotransmission.	Henning et al., 2007
Glutamate	STN	*In vivo* rat model	Increased extracellular glutamate levels in SNr	Windels et al., 2003
Glutamate	STN	Human clinical trail	Pontine glutamate levels were lower in PD patients.	Tuura et al., 2018
GABA	STN	*In vivo* rat model	Increased GABA levels in the SNr.	Windels et al., 2000
GABA	STN	*In vivo* rat model	Increased GABA levels in the SNr.	Savasta et al., 2002
GABA	STN	*In vivo* rat model	Increased GABA levels in the GP.	Salin et al., 2002
GABA	STN	Human clinical trail	Basal ganglia GABA levels were higher in PD patient.	Tuura et al., 2018
GABA	GPi	Human clinical trail	CSF GABA levels increased during stimulation.	Ogura et al., 2004
GABA	GPi	Human clinical trail	Both STN-DBS and Levodopa reduced GABA levels in the central anterior thalamic nucleus.	Stefani, 2011
Serotonin	STN	*In vivo* rat model	Decreased firing rate of DRN 5-HT neurons and induced depressive-like behavior in both PD and control rat model.	Temel et al., 2007
Serotonin	STN	*In vivo* rat model	Decreased both extracellular level and firing of 5-HT neuron.	Tan et al., 2012
Serotonin	STN	*In vivo* rat model	STN-DBS inhibited 5-HT release in the PFC, HIPP, and striatum.	Navailles et al., 2010
Serotonin	STN	*In vivo* rat model	Firing rates of DRN neurons inhibited by HFS-STN-DBS did not quickly revert to their pre-stimulus firing rates.	Hartung et al., 2011
Serotonin	STN	*In vivo* rat model	Severe serotoninergic dysfunction leads to decreased STN-DBS therapeutic efficiency.	Faggiani et al., 2015
Serotonin	STN	*In vivo* mouse model	STN-DBS inhibited 5-HT DRN neuronal activity and induced loss of 5-HT cell phenotype.	Alosaimi et al., 2022
Acetylcholine	PPN	*In vivo* rat model	DREADD introduced in PPN cholinergic neuron activated residual nigrostriatal dopaminergic neurons and reduced motor symptoms.	Sharma et al., 2020
Acetylcholine	PPN	*In vivo* rat model	PPN-LFS mildly reversed the acetylcholine loss in the ventrolateral thalamic nucleus.	Wen et al., 2015
Acetylcholine	STN/GPi	*In vivo* monkey model	STN-DBS produced a larger suppression of TAN spiking rate than GPi-DBS.	Nakajima et al., 2017
Noradrenaline	STN	*In vivo* rat model	Severe LC noradrenergic dysfunction led decrease STN-DBS therapeutic efficiency.	Faggiani et al., 2015
Noradrenaline	STN	*In vivo* rat model	LC noradrenergic degeneration led to weight loss and STN-DBS regained the weight.	Guimarães et al., 2013
Noradrenaline	STN	Human clinical trail	Clonidine manipulation of LC noradrenergic system inhibit the therapeutic effect of STN-DB.	Albares et al., 2015
Noradrenaline	STN	Human clinical trail	Metoprolol manipulation of LC noradrenergic reduce the STN spiking activity in PD patient and improve rigidity.	Coenen et al., 2008

5-HT, 5-hydroxytryptamine (serotonin); 6-OHDA, 6-hydroxydopamine; Ach, acetylcholine; CSF, cerebrospinal fluid; DBS, deep brain stimulation; DA, dopamine; DAT, dopamine transporter; DR1, DR2, and DR3, dopamine 1, 2, and 3 receptors; DREAD, designer receptors exclusively activated by designer drugs; DRN, dorsal raphe nucleus; GABA, γ-aminobutyric acid; GPi, globus pallidus internal; HIPP, hippocampus; LC, locus coeruleus; LFS, low-frequency stimulation; MRS, magnetic resonance spectroscopy; NT, neurotransmitter; NA, noradrenaline; PET, positron emission tomography; PFC, prefrontal cortex; PD, Parkinson’s disease; PPN, pedunculopontine nucleus; RacloBP, [11C] raclopride binding potential; SNc, substantial nigra pars compacta; SNr, substantial nigra pars reticulata; STN, subthalamic nucleus; TAN, tonically active neurons; VMAT2, vesicular monoamine transporter 2.

### Dopaminergic system

Parkinson’s disease is principally defined as pathological degeneration of dopaminergic SNc neurons; newly diagnosed PD patients can show a 50% loss of SNc dopaminergic neurons (Marsden and Parkes, 1977). Moreover, the standard medical treatment for PD (drugs like Levodopa) mainly work on dopaminergic receptors, however, long-term use of medical treatments can exhibit unwanted side effects. Therefore, it has been proposed one mechanism of DBS for PD is its action on surviving striatal DA neurons (Khaindrava et al., 2011). Several early studies were based on this assumption, with some even attempting to directly stimulate the SNc (Preston et al., 1981; Vaccarino and Franklin, 1982; Lin and Yang, 1994). Overtime, STN-DBS showed to improve the motor symptoms of PD more effectively and reduce the need for Levodopa, eventually leading to fewer adverse drug effects (Karl et al., 2022).

Nigrostriatal dopaminergic neurons were assessed in several preclinical animal studies and showed that STN-DBS modulate SNc dopaminergic neurons. Sahai et al. (2020) showed that STN-DBS decreases the spiking activity in less than half (43%) of the SNc dopaminergic neurons in naïve rats group while increasing the spiking activity in the other 43% of the cells. However, the PD rat group showed a significant reduction in the spiking activity of 88% of dopaminergic cells (Sahai et al., 2020). Moreover, an *in vivo* microdialysis study on unilateral 6-OHDA rats showed short-term effects of stimulation and lesioning in the STN increased extracellular levels of dopamine in the striatum (Walker et al., 2009). In another microdialysis study in a freely moving rat model of PD (6-OHDA), STN-DBS increased striatal DA metabolites in awake, freely moving animals (Meissner et al., 2001, 2002). When *in vivo* real-time electrical and chemical detection of dopamine concentrations and neural firings in the caudate-putamen (CPU) of PD rats PD were assessed using fast-scan cyclic voltammetry (FSCV), dopamine concentrations increased and striatal neurons firing decreased following GPi-DBS (Xiao et al., 2019). Additionally, *in vivo* experiments in pigs using a Medtronic 3,389 device to perform FSCV combined with a carbon-fiber microelectrode (CFM) in the striatum to track dopamine release evoked by electrical stimulation showed STN-DBS elicited a stimulus-time-locked increase in striatal dopamine release that was both stimulus intensity- and frequency-dependent (Shon et al., 2010). Another experiment in monkeys showed STN-DBS induced phasic DA release in the striatum (Nakajima et al., 2017).

Further animal experimental studies have investigated the STN-DBS neurotherapeutic effects in improving motor symptoms caused by dopamine in non-human primates and in PD rodent models (He et al., 2014; Yamamoto et al., 2014). One study of MPTP rhesus monkeys treated with STN-DBS and microdialysis implanted bilaterally in the putamen and caudate nuclei showed improvement in motor symptoms and increased extracellular DA along with its metabolites in both brain regions (Zhao et al., 2009). Other researchers used multi-contact DBS electrode, fMRI and FSCV to identify optimum dopamine-recording sites in rhesus macaques and found dopamine release reduces or increases depending on the slight redirection of DBS electrode tip location in the STN while the highest evoked response was shown when the DBS electrode tip contact were in the dorsal part of STN (Min et al., 2016). Lastly, striatal dopaminergic receptors expression was assessed in intact and total nigrostriatal dopaminergic denervated rats after a 4-h unilateral STN-HFS using particular radioligands ([^3^H] SCH 23390, [^125^I] iodosulpride, and [^125^I] OH-PIPAT) and showed increased D1 receptor (D1R) binding in all areas of the striatum but decreased binding of both D2 and D3, leading some to suggest that D1R effects could explain the therapeutic effect of DBS while side effects could be more due to D2R and D3R (Carcenac et al., 2015; Melon et al., 2015).

Some evidence suggests that STN-DBS for PD has a long-term neuroprotective effect on the survival of SNc cells in animal models (Temel et al., 2006; Wallace et al., 2007; Spieles-Engemann et al., 2011). Khaindrava et al. (2011) investigated DBS of the STN and found no significant increase in cell proliferation, yet cell survival in dopaminergic neurons of the striatum was increased. In addition, lesser amounts of cell proliferation was observed in the hippocampus and olfactory bulb. Another study investigated the neuroprotective effects of STN-DBS on a unilateral 6-OHDA model in rats; post-stimulation effects revealed that nigral TH positive neurons and the protein phosphatase-2A (PP2A) were blocked inducing autophagy and dissociating the Bcl-2/Beclin1 complex showing a possible neuroprotective mechanism for PD (Du et al., 2018). Another STN-DBS study in 6-OHDA rat model of PD showed BDNF-mediated neuroprotection in the SNc by acute and long-term blocking of TrkB enzyme that decreased phosphorylation of Akt and ribosomal protein S6 (Fischer et al., 2017). Another study investigated the protective effect of DBS in the STN in unilateral 6-OHDA rats and found apoptosis significantly decreased in the DBS group.

Some clinical findings toward the effects of DBS on striatal dopaminergic neurons have shown to be contradictory. A PET study in PD patients assessed vesicular monoamine transporter 2 (VMAT2) and cerebral glucose metabolism before DBS surgery and 4–6 months after STN-DBS and showed improvement of motor and neuropsychiatric symptoms while VMAT2 had decreased in the caudate and putamen along with decreased cerebral glucose metabolism in the striatum (Smith et al., 2019). Another PET study showed synaptic dopamine concentrations increase in the putamen and caudate nucleus after STN-DBS alongside medication; Levodopa significantly reduced [11C] raclopride binding potential (RacloBP) in the putamen, while post-operatively the reduced RacloBP-binding reversed (Nimura et al., 2005) and the drug-induced increase in synaptic dopamine concentrations was also higher after stimulation.

A clinical study used SPECT to assess the dopamine D2 receptor and dopamine transporter (DAT) in PD patients pre-surgery and at 12 months post-surgery; the unified PD rating scale (UPDRS) scores post-surgery remarkably improved in those patients and titrated down their medication, but no changes in DAT availability or an increase in D2 receptor binding were detected (Hesse et al., 2008). Another clinical study investigated the occurrence of apathy and depression following 12-month STN-DBS in PD and control groups and found that PD exhibited more apathy and depression symptoms. Additionally, sections of each group received a PET-scan ([11C]-raclopride) that showed increased binding of D2/D3 receptor density. In sum, these findings reflect that decreased synaptic dopamine levels in the mesolimbic area, particularly the ventral tegmental area (VTA) are more substantial in PD patients, suggesting that apathy and depression occur post-surgery as a postponed dopamine withdrawal syndrome (Thobois et al., 2010).

Clinical PET scans in 6 PD patients receiving STN-DBS showed no difference in RACLO binding (ligand for dopamine D2/D3 receptor) between DBS on and off conditions along with no evidence of increased striatal dopamine concentration under effective STN-DBS (Hilker et al., 2003; Strafella et al., 2003). In another clinical trial, cerebrospinal fluid and plasma catecholamine levels in STN-DBS treated PD patients were measured after oral antiparkinsonian drug administration before surgery and an hour after medication while being on DBS; higher pre-operative catecholamine levels were linked to better STN-DBS outcomes (Yamamoto et al., 2015).

Stereotactic microdialysis is feasible in PD patients during STN-DBS surgery (Kilpatrick et al., 2010; Zsigmond et al., 2012). Within 30 min of DBS electrode placement in the STN and a microdialysis probe in the STN or SN researchers can get a steady-state baseline level of glutamate, dopamine, and GABA (Kilpatrick et al., 2010). A microdialysis study in the GPi during GPi-DBS surgery investigated dopamine levels before and after DBS and found that 4 out of 5 patients had significantly increased pallidal DA after stimulation (Martinez et al., 2013). However, there was no association between the improvement in rigidity and pallidal DA increase, suggesting other mechanisms might be involved in these clinical effects of GPi-DBS (Martinez et al., 2013).

In conclusion, the dopaminergic system is essential in PD as is clearly mediated with pharmaceutical and DBS therapies. DBS has shown to treat motor symptoms of PD and decrease the need for DA medication, suggesting DBS has a synergetic effect on DA. Preclinical animal data suggests that STN DBS influences both local and remote dopaminergic systems, while clinical microdialysis data in human subjects point more toward a local effect. Remote effects are less consistent in imaging studies, as inconsistencies stem from limitations innate to PET and SPECT neuroimaging techniques.

### Glutamatergic system

Glutamatergic neurons are distributed throughout the central nervous system (Greenamyre, 2001). Several theories suggest overactive glutamate in the basal ganglia induces excitotoxin production in SNc neurons contributing to the pathogenesis of PD (Olney et al., 1975; Piallat et al., 1996; Saji et al., 1996; Smith et al., 1996; Rodriguez et al., 1998; Nakao et al., 1999). An experimental stereotactic injection of glutamate receptors antagonist (NMDA blocker) in the striatum of rodents has shown to alleviate parkinsonian symptoms (Carroll et al., 1995; Schmidt, 1995). It then appears that DBS could influence the glutamatergic system in a net positive way as neurons within the striatum act as intermediate excitatory neurons between basal ganglia-thalamocortical circuits (Greenamyre, 2001).

Several pieces of evidence suggest that DBS effects on glutamate is solely a local effect that only lasts during stimulation. In an experimental study of anesthetized rats, a dual enzyme-based electrochemical sensor measured extracellular glutamate concentrations in the STN and found that glutamate concentrations increased rapidly during DBS and were sustained during stimulation. After cessation of stimulation, elevated glutamate levels slowly fell toward baseline (Lee et al., 2007). Another study assessed the dyskinesia side-effects of STN-DBS in unilateral 6-OHDA rats using microdialysis in the SNr; STN-DBS increased glutamate and induced dyskinesia in both intact and hemiparkinsonian rats (Boulet et al., 2006). When the stimulation frequency was lowered, glutamate levels were unaffected suggesting dyskinesia blocks glutamate receptor antagonists and facilitates agonists. In another study, STN-DBS was performed in rats to determine if neurochemical changes in the GP and SNr were frequency-dependent and found that glutamate concentrations were significantly increased at a high frequency (60 and 130 Hz) in the GP and SNr but did not show significant change at LFS (10 Hz) (Windels et al., 2000, 2003). It should be noted that the measured glutamate levels in the GP refers only to the GPe, and is not a basal ganglia’s output structure unlike the SNr. The entopeduncular (EP) nucleus is homologous to the primate GPi in rats (Karain et al., 2015).

Chassain et al. (2016) studied glutamate metabolic, synaptic, and behavioral changes in hemiparkinsonian rats after 5 weeks of chronic STN-DBS using proton magnetic resonance spectroscopy (11.7T) and found chronic STN-DBS corrects the glutamate metabolites levels associated with neurotransmission in the striatum and SNr, restores corticostriatal synaptic plasticity, and restores motor skills progressively in the staircase test. Consequently, these findings suggest chronic STN-DBS not only has a local effect but also a remote effect in the basal ganglia. Furthermore, STN-DBS showed a reduction in sensitivity toward glutamate neurotransmission via downregulating calcium/calmodulin-dependent kinase IIa (CaMKIIa) and Homer1 genes in the STN as these genes are associated with glutamate neurotransmission (Henning et al., 2007). These findings suggest STN-DBS could have a neurotherapeutic effect by alleviating overactive glutamatergic neurons in the STN. To validate these findings in humans, clinical studies were performed to assess glutamate levels in PD patients and controls using magnetic resonance spectroscopy. Pontine glutamate levels were shown to be lower in PD patients while glutamate levels also emerged as a significant predictor of outcome, further implementing glutamatergic neurotransmission within the mechanisms of DBS (Tuura et al., 2018).

In sum, the glutamatergic system has shown to be involved in PD pathology. STN-DBS modulates glutamatergic neurons and glutamate neurotransmission and exhibits neurotherapeutic effects by improving motor and adverse effects. Acute effects of STN-DBS on the glutamatergic system were thought to be merely local and stimulation-dependent, however, chronic effects of STN-DBS show increases in glutamate in the striatum and SNr and further demonstrate to improve motor symptoms of PD in preclinical studies.

### GABAergic system

GABAergic neurons are distributed widely through the entire central nervous system (Olsen and Li, 2012) and act mainly as interneurons, having an essential role in regulating cortical and subcortical circuits including the cortico-thalamic-basal ganglia circuits (Zhang et al., 2021). GABAergic neurons function harmoniously with glutamatergic and dopaminergic neurons in the cortical-thalamic-basal ganglia circuit to control movement. As previously described, glutamatergic neurons have an excitatory effect while GABAergic neurons have inhibitory projections in the basal ganglia. Dopaminergic neurons also have both excitatory and/or inhibitory (depending on momentum) after-effects on glutamatergic and GABAergic modulation balance (Zhang et al., 2021).

Subthalamic nucleus-DBS has shown to increase the neuronal firing rate in the SNr of PD rats (Benazzouz and Hallett, 2000). Moreover, preclinical studies show an increased selective extracellular GABA release in the SNr after STN-DBS in rats (Windels et al., 2000; Savasta et al., 2002). This increase of GABA release in the SNr was shown within a stimulation frequency-dependent range of 60 to 350 Hz, while glutamate release only increased until 130 Hz (Windels et al., 2003). STN-DBS could then have both local and cumulative effects on GABA release in the SNr that pass from the STN to the SNr, independent of the STN-DBS effect on glutamate release in the GP (McIntyre et al., 2004). Additionally, *in situ* hybridization of glutamate decarboxylase 67 kDa isoform (GAD67) study also showed an increase in GABA in the GP after STN-DBS in unilateral 6-OHDA rats (Salin et al., 2002).

Clinical studies demonstrate GPi-DBS to improve bradykinesia and Levodopa-induced dyskinesia (LID) in PD patients through its effects on pallidum GABAergic neurons (Obeso et al., 2000; Calon and Di Paolo, 2002). Moreover, depending on the specific stimulation site within the GPi it was found that DBS of the ventral GPi reduced LID while stimulation of the dorsal part showed to improve bradykinesia (Krack et al., 1998; Vitek et al., 2004).

Using microdialysis in the GPi and ventral anterior (VA) thalamus during the first delivery of STN-DBS or Levodopa, researchers found both treatments reduced GABA levels in the VA thalamus, while STN-DBS also increased cyclic guanosine monophosphate (cGMP) levels in the GPi (Stefani, 2011; Stefani et al., 2011). A clinical imaging study assessing GABA levels of PD patients and controls using magnetic resonance spectroscopy showed basal ganglia GABA levels to be higher in PD patients (Tuura et al., 2018). In another clinical study on neurotransmitter levels in CSF of PD patients with GPi-DBS, CSF was collected a day after surgery 1 h before and 1 h after GPi stimulation. GABA levels increased during stimulation but no differences were seen in levels of dopamine, noradrenaline, or homovanillic acid (Ogura et al., 2004).

In conclusion, it is clear that GABAergic interneurons play a critical role in movement regulation and within the dynamics of STN- and GPI-DBS. However, GPi-DBS effects depend on whether the ventral or dorsal part of the GPi is stimulated (Krack et al., 1998; Obeso et al., 2000; Calon and Di Paolo, 2002; Vitek et al., 2004). Furthermore, local impacts of STN-DBS show an increase in GABA in the GP while remote effects connected to the SNr are low to high frequency stimulation-dependent and differ from glutamate responses (Windels et al., 2000; Salin et al., 2002; Savasta et al., 2002).

### Serotoninergic system

The serotonergic system is well known to be involved in mood and anxiety (Smith et al., 1997), while patients who undergo STN-DBS have shown side effects related to serotoninergic and dopaminergic systems such as depression, suicide ideation, and impulsivity (Voon et al., 2008; Temel et al., 2009; Thobois et al., 2010; Tan et al., 2011a). Several lines of evidence show that STN-DBS inhibits the serotoninergic system, and while the STN has no direct connection to the DRN, its relay station via the lateral habenula (LHb) suggests an indirect serotonin-modulating mechanism (Tan et al., 2011b).

Studies have shown that bilateral STN-DBS reduces the firing rate of DRN serotonergic neurons and induces depressive-like behavior in both PD and control rat models (Temel et al., 2007). Furthermore, the firing rate of DRN neurons that were inhibited by STN-DBS did not quickly recover to their pre-stimulus firing rates; many of these neurons remained to show reduced activity throughout the 5-min post-stimulus recording period. This suggests STN-DBS elicits mechanisms that may cause sustained suppression of the serotonergic system (Hartung et al., 2011). Moreover, STN-DBS inhibited 5-HT release in forebrain regions such as the prefrontal cortex (PFC), hippocampus (HIPP), and striatum (Navailles et al., 2010; Tan et al., 2012). Recent research shows that long-term (10 weeks) STN-DBS inhibits the serotoninergic neuronal activity during stimulation measured by calcium transients photometry and leads to a loss of serotoninergic cell phenotypes inducing depressive-like behavior in the MPTP mouse model of PD (Alosaimi et al., 2022). Animal studies also show that presence of severe serotoninergic dysfunction reduces STN-DBS therapeutic efficiency (Faggiani et al., 2015). In addition, the 5-HT system has been implicated in dyskinesia (Sandler, 1996; Lauterbach and Shillcutt, 1997; Caley, 1998; Kapur et al., 1999; Creed-Carson et al., 2011). However, it can be speculated that a sustained suppression of the 5-HT system via loss of 5-HT cell phenotype could contribute to the lower incidence of dyskinesia following STN-DBS. In the other words, reduced basal ganglia 5-HT function supports the therapeutic effects of DBS. Future research is needed to explore the exact trajectory connections between the STN and the DRN that engender DBS targeting to display fewer adverse effects.

### Cholinergic system

Neurons of the cholinergic system are predominantly located in the nucleus basalis of Meynert, the PPN, and striatum (Mesulam and Geula, 1988; Heckers et al., 1992; Mesulam et al., 1992). Recent data suggests the degeneration of cholinergic neurons could be involved in the pathogenesis of early stage PD and linked to its axial and non-motor symptoms, including cognitive decline and mood disorder (Mayeux et al., 1981; Green et al., 2002; Braak et al., 2003; Aarsland and Kurz, 2010; Wilkins et al., 2020). Moreover, striatal cholinergic interneurons regulate basal ganglia circuits and could modulate effects of dopaminergic and glutamatergic systems (Liu, 2020).

Animal studies have investigated tonically active neurons (TANs) and putative cholinergic interneurons in the striatum during DBS; STN-DBS produced a more extensive suppression of TAN spiking rates compared to GPi-DBS in healthy monkeys, additionally, a local DA antagonist infusion in the striatum only reduced the spike rate after STN-DBS, but not in the GPi-DBS group (Nakajima et al., 2017). This suggests a more apparent increase of DA after STN-DBS antagonized by the local DA infusion and counterbalanced by the putative cholinergic interneurons.

Pedunculopontine nucleus-DBS has been proposed to treat axial and gait symptoms of PD. PPN-LFS (25 Hz) in unilateral 6-OHA rats improved gait symptoms including base of support and maximum contact area in the catwalk test assessing locomotion and gait function (Wen et al., 2015). Furthermore, PPN-LFS also mildly reversed acetylcholine loss in the ventrolateral thalamic nucleus in rodents (Wen et al., 2015). Sharma et al. (2020) investigated the stimulation effects of designer receptors exclusively activated by designer drugs in the PPN using a PET scan to assess nigrostriatal dopaminergic neurons in a chemogenetic PD model and found that residual nigrostriatal dopaminergic neurons reduced motor symptoms (Sharma et al., 2020). PPN-HFS has also shown to reduce postural instability of PD-rats (6-OHDA), however, rats stimulated in the PPN showed complex behavior effects (Rauch et al., 2010). In sum, the cholinergic system regulates motor functions in the basal ganglia and is associated with axial and gait symptoms in PD. Preclinical animal studies of PPN-DBS exhibits an improvement in axial and gait symptoms however the data is mixed. It remains to be determined whether the PPN is a suitable new stimulation target for patients with severe axial and gait dysfunction.

### Noradrenergic system

Noradrenaline (NA) is predominantly produced by the LC located in the pons and projects to cortical, subcortical, and spinal structures (Tubbs et al., 2011). Noradrenergic LC neurons have shown to be involved in a wide range of sensory-motor, behavioral, and cognitive functions (Foote et al., 1980, 1983; Aston-Jones, 2005; Benarroch, 2009; Berridge et al., 2012; Uematsu et al., 2015; Feinstein et al., 2016; Breton-Provencher et al., 2021). Evidence suggests the loss of NA neurons occurs several years prior to presentation of PD symptoms and more extensively than in SNc dopaminergic neurons (Zarow et al., 2003; Rommelfanger and Weinshenker, 2007; McMillan et al., 2011; Espay et al., 2014). Additionally, PD exhibits a neuropathological vulnerability to neuromelanin loss that is shown to affect both DA in the SNc and NA in the LC (Hirsch et al., 1988; Sasaki et al., 2006; Ohtsuka et al., 2013; Martin-Bastida et al., 2017; Singh, 2020).

In recent years, the effects of DBS on LC noradrenergic system have gained considerable attention. A preclinical animal study showed that severe noradrenergic dysfunction reduces STN-DBS therapeutic efficiency within a PD rat model (Faggiani et al., 2015). Furthermore, Guimarães et al. (2013) investigated the involvement of the LC noradrenergic system on weight loss in a PD rat model and showed STN-DBS abolished weight loss in bilateral LC and striatum 6-OHDA lesioned rat without any observed changes in their food or other metabolic parameters. Additionally, the degeneration of the LC was not accompanied by significant changes in motor behavior but led to an extra decrease in striatal monoamine levels reflected by the decrease in the DA/L-3,4-dihydroxyphenylalanine (L-DOPA) ratio (Guimarães et al., 2013).

A clinical study suggests a role for the LC noradrenergic system in STN-DBS. When stimulation is combined with administration of clonidine, a selective alpha2 adrenergic agonist, STN-DBS related benefits on akinesia are diminished (Albares et al., 2015). On the other hand, one clinical study showed that metoprolol, a beta1-adrenergic antagonist, suppressed STN bursting activities marking a brief decrease in rigidity pre-STN-DBS surgery (Coenen et al., 2008). Retrospective clinical data showed STN-DBS led to weight regain in PD after DBS surgery (Tuite et al., 2005; Strowd et al., 2010) and this was suggested to be related to a STN-DBS effect on the LC noradrenergic system (Guimarães et al., 2012).

In conclusion, LC noradrenergic dysfunction occurs years before clinical diagnosis and shows a similar pathological root to PD comparable to SNc dopaminergic systems. Furthermore, severe dysfunction of the noradrenergic system diminishes positive effects of STN-DBS, as STN-DBS has shown to improve weight loss in PD possibly through its actions on the LC noradrenergic system. Pharmacological manipulations of the noradrenergic system further impact PD symptoms even alongside STN-DBS and this underlines the need for research combining pharmacological and DBS treatments.

## The role of glia cells in neurotransmitter homeostasis

Glia cells, including astrocytes and microglia, are involved in inflammatory processes and contribute to neurodegeneration of the SNc in PD, as previously reviewed by McGeer and McGeer (2004, 2008). In addition, astrocytes have a role in neural communication and exhibit effects on neuronal activities (Mennerick and Zorumski, 1994; Pfrieger and Barres, 1997). For instance, astrocytes can store and release glutamate stimulating pre- and post-synaptic receptors of surrounding neurons (Norenberg, 1979). Consequently, inflammatory processes activate astrocytes in the SNc to release glutamate which project to the STN and can lead to glutamatergic neuron overactivity. This glutamatergic overactivity has also been shown to promote microglia and pro-inflammatory cytokines release that can contribute further to SNc neuronal damage (Brochard et al., 2008). Additionally, inflammatory processes can enhance α-synuclein release linked to glutamatergic excitotoxicity in the SNc (Gu et al., 2010; Sarafian et al., 2017).

Deep brain stimulation has shown to affect glia cells even starting from an initial reaction to the implantation of a DBS electrode (DiLorenzo et al., 2010). Although the glia cells cannot generate action potentials, their cellular properties allow sensitivity to voltage changes possibly including external electrical stimulation (Marrero et al., 1989; Barres et al., 1990). This electrical stimulation mainly activates astrocytes via intracellular calcium released locally on the stimulation site and to surrounding cells (Cornell-Bell and Finkbeiner, 1991; Pasti et al., 1997; Wang et al., 2006; Shigetomi et al., 2008). Moreover, calcium propagation to astrocytes could induce glutamate neurotransmission (Cornell-Bell et al., 1990; Angulo et al., 2004). DBS has been further indicated to modulate different subtypes of glia cells, including astrocytes, microglia, and macrophages that can change glial phenotypes and their functions contributing to therapeutic effects (Vedam-Mai et al., 2012). A recent experimental animal study showed STN-DBS inhibits neuroinflammatory processes in PD by modulating glia cells including astrocytes in the GP, shown in both *in vivo* rat models and *in vitro* cell cultures (Campos et al., 2020). Another experimental study showed STN-DBS inhibits microglia and normalizes neuroinflammatory cytokine levels in the SN of a rat PD model (Chen et al., 2020). As a consequence, glia cells involved in both PD and STN-DBS modulate neuroinflammatory processes, while a greater understanding of the underlying mechanisms could improve therapeutic outcomes involving the survival of SNc dopaminergic and STN glutamatergic neurons (Collier and Sortwell, 1999).

## Conclusion

It has been shown that multiple neurotransmitters are involved in the neuropathology and pathophysiology of PD, including the SNc dopaminergic, LC noradrenergic, pallidal GABAergic, PPN cholinergic, DRN serotoninergic, and STN glutamatergic systems. Growing evidence supports glia cells, especially astrocytes, to be involved in PD as well, while DBS modulates these cells by mainly effecting glutamatergic neurons (Brochard et al., 2008). Although DBS improves motor symptoms in PD and continued evidence shows underlying neurochemical alterations involved in PD can be mediated by DBS, the exact mechanisms of action underlying DBS effects on neurotransmitters are still not fully understood.

Deep brain stimulation has demonstrated alleviation of motor symptoms of PD while decreasing the need for pharmaceutical dopamine treatment, indicating DBS impacts dopaminergic system. In preclinical research, STN-DBS has both local and distant effects on dopaminergic systems (Meissner et al., 2002; Walker et al., 2009; He et al., 2014; Yamamoto et al., 2014; Carcenac et al., 2015; Melon et al., 2015; Min et al., 2016; Nakajima et al., 2017; Du et al., 2018; Sahai et al., 2020). In contrast, clinical results demonstrate local impacts from STN-DBS using electrophysiological and microdialysis, but such data shows less consistency compared to distant effect from neuroimaging studies (Hilker et al., 2003; Strafella et al., 2003; Nimura et al., 2005; Hesse et al., 2008; Thobois et al., 2010; Yamamoto et al., 2015). Moreover, STN- and GPi-DBS affects glutamatergic neurons and glutamate neurotransmitter release leading to side effects, mainly dyskinesia (Boulet et al., 2006). The acute effects of DBS on the glutamatergic system were once assumed to be local and stimulus-dependent, but preclinical investigations of prolonged DBS have shown to increase glutamate in distant areas including the striatum and the SNr (Windels et al., 2000, 2003). DBS effects on the GABAergic system also differ based on specific DBS target; STN-DBS has local influence on GABA in the GPi and a distant effect on the SNr where it is more stimulation-dependent (Benazzouz and Hallett, 2000; Johnson and McIntyre, 2008). GPi-DBS also has local effects on GABA release in the GPi and remote effects in the SNr, however, ventral GPi-DBS shows alleviation in LID whereas dorsal GPi-DBS reduces bradykinesia symptoms (Krack et al., 1998; Vitek et al., 2004). STN-DBS has demonstrated superiority in treating bradykinesia and reducing Levodopa requirements (Williams et al., 2014).

The frequent occurrence of depression among PD patients suggests a role for the DRN serotoninergic system. This is stressed by STN-DBS induced inhibition in serotoninergic neurons in PD. From preclinical data, PPN-DBS has shown to improve axial symptoms in PD, although further research is needed before clinical translation can move forward (Wen et al., 2015; Sharma et al., 2020). Lastly, noradrenergic systems have recently driven attention there way as the degeneration of the LC noradrenergic system has shown to lead to DBS therapy resistance (Faggiani et al., 2015). Combined pharmacological-DBS treatments should continue be a focus in future research to investigate if other neurotransmitters including the cannabinoid and opioidergic systems may also prove to be involved in mechanistic symptom mediation (Turner et al., 2017; Han et al., 2018).

One reason neurochemical changes from DBS are not extensively covered within the literature could be from technical limitations innate to detecting transmitter release and transmitter-related charges in remote neuronal areas. Optogenetic studies investigating specific effects of neuromodulation on neurotransmitter release are warranted as they would further help assess DBS cumulative and chronic effects on local and remote neural elements. Further understanding dynamic changes in neurotransmitters will work to improve DBS effectiveness, provide more precise targeting, reduce adverse effects, and provide more appropriate pharmacological intervention options to improve overall quality of PD treatment.

## Author contributions

FA: writing–original draft preparation, editing, and visualization. JB: writing–review, editing, proofreading, and visualization. ST: writing–review and editing. YT: writing–review. AJ: conceptualization, writing–review, and editing. All authors contributed to the article and approved the submitted version.
